# Check with the Intended Audience First! Content Validation as a Method for Inclusive Research for Primary Care Engagement in Rural Appalachia

**DOI:** 10.13023/jah.0601.06

**Published:** 2024-09-01

**Authors:** Sydeena E. Isaacs, Jennifer Schroeder Tyson, Ashley Parks, Danielle Adams

**Affiliations:** Appalachian State University; Appalachian State University; Appalachian State University; Appalachian State University

**Keywords:** Appalachia, content validation, primary care engagement survey, rural health

## Abstract

**Introduction:**

To date, referral practices based on social determinants of health (SDOH) among primary care providers (PCPs) and clinic staff in rural regions, including Appalachian North Carolina (NC), are not well understood.

**Purpose:**

This study aims to develop and content validate a primary care engagement (PCE) survey to assess (1) engagement and burnout; (2) referral practices; and (3) self-efficacy and confidence in making referrals based on SDOH among PCPs and clinic staff in Appalachian NC.

**Methods:**

Using the Social-Ecological Model as a theoretical framework, researchers developed a 37-item PCE survey. Content validation was completed by a panel of experts recruited from a convenience sample of faculty at a local university and PCPs and clinic staff practicing in Appalachian NC. Participants rated the degree of relevance of survey questions on a four-point Likert scale and provided additional feedback about the wording/appropriateness for the intended audience. Content validity index (CVI) scores were calculated for each question by averaging the degree of relevance ratings.

**Results:**

Ten participants completed the study between August and November 2022 (nurse practitioners, academic researchers, clinical support staff/quality improvement associates, administrative staff supervisor, administrator/practice manager). CVI scores for each item ranged from 3.43 to 4.0. Comments regarding potential improvements were primarily focused on small edits, including grammar-related changes and opportunities for clarity and inclusivity.

**Implications:**

High CVI ratings for all survey items indicate the overall approach/survey aim resonates with local clinicians and individuals with expertise in SDOH. This study and the final survey lay the foundation for collaborative, collective-impact initiatives that are directly informed by the survey findings.

## INTRODUCTION

Rural populations are disproportionately affected by many health inequities compared to the rest of the nation, including a higher incidence of chronic diseases, higher mortality rates, and lower life expectancies.^1^ The Appalachian Region, also known as Appalachia, is one of the rural regions that experiences both significant socioeconomic and health inequalities compared to the rest of the nation.^2^ The AR has higher mortality rates for seven of the leading causes of death nationwide, including heart disease, stroke, cancer, and diabetes.^2^

Understanding access to primary healthcare services in a community is important, as there is extensive evidence that having a primary medical home and access to wellness screenings and health care to prevent, treat, and manage disease helps support better health outcomes.^3^ Having a medical home and access to care also lowers healthcare costs in the community overall through the reduction of both emergency services utilization and the occurrence of preventable health conditions.^4^ In Appalachia, the supply of primary care and mental health providers, specialty physicians, and dentists is significantly lower compared to national averages (20%, 35%, 28%, and 26%, respectively)^2^; rural counties within the region are most impacted by these disparities. Several parts of Appalachia, including Western North Carolina (WNC), the focus of the current study, are designated as medically underserved.^5^ This means there is a lack of primary care providers (PCPs), high infant mortality rates, a high elderly population, or a high poverty rate.^6^ The major factors for the lack of access to health care are low income, geographic isolation, and poor knowledge of available programs. The lack of healthcare facilities in the area, combined with its isolation, means it can take more than an hour for some families to reach a hospital.^7^ Due to this lack of access, people may often go long periods of time without visiting a healthcare professional for checkups because they do not feel ill. This allows diseases without highly uncomfortable initial symptoms (e.g., hypertension and diabetes) to worsen.^8^–^10^

Social determinants of health (SDOH) are the non-medical conditions in which people are born, live, grow, and work.^11^ Adverse SDOH (e.g., food insecurity, unemployment, and housing insecurity) are strong determinants of poor health outcomes, such as risk for diabetes,^12^ cardiovascular disease,^13^ and hypertension. Referrals to services based on SDOH are a critical component of strengthening health systems through the mitigation of unmet health-related social needs (e.g., education, unemployment, food insecurity, housing insecurity, and mental health).^14^ However, utilization of some community and federally funded social programs (e.g., WIC and SNAP) is lacking. Research suggests lack of referrals to these services may contribute to the lack of participation.^15^ As such, understanding provider referral practices and perceptions of and self-efficacy for making referrals is imperative to identify potential referral barriers and intervention foci.

A recent scoping review by Quiñones-Rivera et. al^16^ explored health care providers’ knowledge, attitudes, beliefs, and behaviors regarding socioeconomic risk (e.g. food insecurity, unstable housing, transportation, or health literacy) screening and referral interventions. Providers included primary care physicians, resident physicians, nurses, nurse practitioners, social workers, certified health workers, pharmacists, and others. Results indicated that while most providers expressed positive perceptions and attitudes towards addressing patients’ socioeconomic risks, they also had concerns regarding insufficient knowledge and resources, discomfort with screening, time and workflow disruptions, and potential negative impacts of screening and referral programs on relationships with patients. Yet most studies of this nature have been conducted in urban, pediatric, and/or large medical centers. To date, referral practices based on SDOH among PCPs and clinic staff in rural regions, including Appalachian NC, are not well understood.

A formative, qualitative study by Isaacs et. al^15^ examined barriers and facilitators to WIC program participation among pregnant women and mothers in rural Appalachian NC. Remarkably, none of the participants in the study were made aware of the program by a PCP or obstetrician. Furthermore, many were unaware that pregnant women qualify for WIC services. These findings outline the need to better understand provider referral practices and perceptions of and self-efficacy for making referrals based on SDOH in rural Appalachian NC. However, to fully explore the factors that impact referral practices, it is also important to consider the pressures providers and staff are facing in the context of a recent global pandemic and the overwhelming prevalence of provider burnout^17^ that could impact self-efficacy and engagement. Therefore, this study aims to develop and content validate a primary care engagement (PCE) survey to assess engagement and burnout, referral practices, and self-efficacy and confidence in making referrals based on SDOH among PCPs and clinic staff in Appalachian NC. This research study is the first of its kind to focus on the rural AR.

## METHODS

### Theoretical Basis and Survey Development

**Figure 1:**
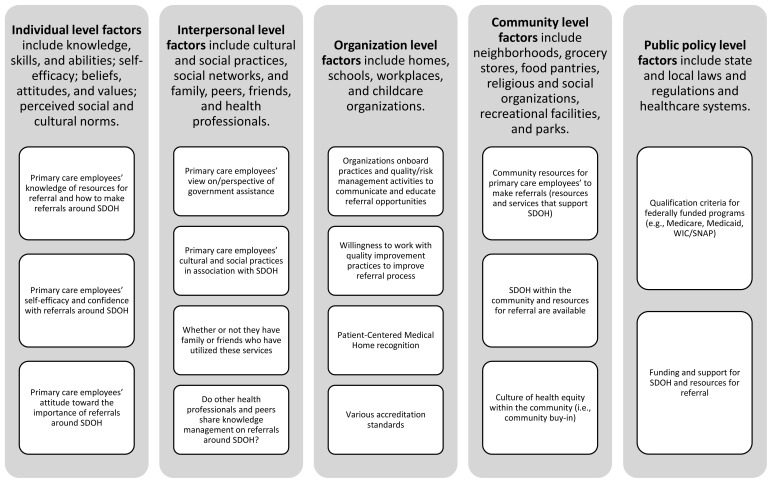
Social-Ecological Model Theoretical Framework Applied to Referrals Based on SDOH

Following an extensive literature review, the research team developed a 37-item survey using the Social-Ecological Model (SEM)^18^ as a theoretical framework (Fig. 1). The SEM posits that behavior change occurs across five interconnected levels: individual, interpersonal/social environment, organizational, community, and public policy.^18^ Survey questions target individual- and interpersonal-level factors. Twenty-seven survey items were adapted from two previously validated instruments: the Mini Z Survey^17^ and the PS#90.^19^,^20^ The Mini Z is a 10-item, validated instrument from the Institute for Professional Worklife that measures engagement and burnout among healthcare providers and staff members.^21^ Sample questions include *“Overall, I am satisfied with my current job”* and *“My control over my workload is”*. Responses are interpreted on five-point Likert scales ranging from “*agree strongly*” (5) to “*strongly disagree*” (1) and from “*optimal*” (5) to “*poor*” (1).

The PS#90 is a 38-item instrument from the American Academy of Pediatrics that collects information on providers’ professional development activities and practices when caring for low-income children.^19^ Responses are interpreted on four-point Likert scales for questions assessing the frequency of, or comfort level with, performing a specified task or knowledge of available community resources (i.e., *“1 = almost never or <25% of visits”* to “*4 = almost always or ≥75% of visits”*) and a five-point Likert scale for assessing level of agreement with a specified statement or perception of a specific community resource (i.e., *“1 = strongly disagree”* to *“5 = strongly agree.”)* Seventeen items from the PS#90 were adapted for use in the current study. An additional 10 items were developed by the research team. Eight items assessed socio-demographic characteristics of survey participants (i.e., race, ethnicity, gender, sexual orientation, county of practice, facilities/clinics where practices, position at the clinic, and length of time working at organization). Two additional items asked if participants are involved in referral services for patients/clients (response options “*yes*” or “*no*”) and if they have observed any way to improve referrals to other community services based on SDOH (responses interpreted on a five-point Likert scale from “*definitely yes”* to “*definitely not”*).

### Content Validation

Content validation is the assessment of how well the items in a survey instrument measure the expected constructs of interest. More specifically, content validity measures the relevance, representativeness, and technical quality of a survey tool.^22^ Content validity is often assessed by a panel of experts including academics and practitioners who have expertise from their field or industry and are familiar with (or part of) the target population for the survey. Experts in survey development are also commonly utilized to assess structural aspects of the survey such as confusing or leading questions. By including the intended audience in survey content validation, the power dynamic shifts from “*us v. them*” to “*we*” and ensures diverse perspectives and experiences are represented.

### Participants

The target population for the final PCE survey is actively practicing PCPs and clinic staff involved in making referrals to services based on SDOH in the 31 AR counties of western NC. Per the NC Institute of Medicine, PCPs are defined as physicians, nurse practitioners, physician assistants, and certified nurse-midwives. 23 For this study, clinic staff were defined as registered nurses, certified nursing assistants, administrative assistants/clinical support staff, or any other staff member involved in making referrals to services based on SDOH.

Content validation of the survey instrument was completed by experts with related experience, direct knowledge of the target population, and/or related research expertise in survey development, rural health, SDOH, and similar topics. Eligibility criteria included the following: (1) at least 18 years of age and (2) identify as either (a) a PCP who is actively practicing or has previously practiced in a primary care clinic in the AR of North Carolina; (b) a current or previous primary care clinic staff member involved in making referrals to services based on SDOH in the AR of NC; or (c) an academic researcher with related expertise, including SDOH, rural health, health disparities, survey design, healthcare quality improvement, etc. The study protocol and procedures were approved by Appalachian State University's Institutional Review Board.

### Study Procedures

Content validation procedures for the current study were adapted from previous research.^22^,^24^ Convenience and snowball sampling were used to recruit eligible participants. A brief recruitment email was sent to all faculty members in the College of Health Sciences (c.150 faculty members) at Appalachian State University and 10 local PCPs and clinic staff members. Those interested in participating were asked to complete a study interest form via Qualtrics through which they were screened for eligibility and asked to provide their email address for follow-up. A second email with a Qualtrics link to the PCE survey validation form, developed specifically for this study, was sent to all eligible participants. The validation form was an exact copy of the PCE survey with two additional validation questions after each survey question. Participants with previous or current experience as a provider or clinic staff member were instructed to answer each survey question based on their experience and then (1) rate the degree of relevance of each question on a four-point Likert scale (responses ranging from *“1 = not relevant”* to *“4 = highly relevant”)* and (2) provide any additional feedback about the wording of the questions and their appropriateness for the intended audience. Relevance refers to the item’s ability to represent the degree of engagement and burnout, referral practices, and self-efficacy and confidence in making referrals to the intended audience. Participants with research expertise and practical experience were instructed to follow the same procedures, except they were not required to respond to the actual survey questions. All data were collected between August to November 2022.

### Data Analysis

Content validity index (CVI)–degree of relevance scores were calculated for each survey item by averaging the relevance scores provided by individual respondents. It was determined a priori that items with a CVI–degree of relevance score less than 3.0 would be removed from the survey. Additional feedback comments and suggested revisions to the wording of each question were compiled, reviewed at length by the research team, summarized into thematic categories utilizing qualitative analysis software, and used to inform the edits required to construct the final iteration of the survey. Specifically, manual open coding methods were utilized to code all comments from the content validation participants in the MAXQDA qualitative analysis software. Following the initial open coding process, content validator responses were grouped and organized across six prominent themes.

## RESULTS

Thirteen participants completed the Qualtrics content validation interest form and were eligible to participate in content validation based on their professional roles and expertise. Of those who were eligible, ten (77%) completed the content validation form, including four nurse practitioners, two academic researchers, two clinical support staff/quality improvement associates, one administrative staff supervisor, and one administrator/practice manager. All content validators rated the degree of relevance for all survey items as 3 or higher, as shown in Table 1 (see subsequent pages). CVI–degree of relevance scores for each item ranged from 3.43 to 4.0. Content analysis of the comments and feedback provided by respondents revealed six major themes, which are described below. Table 2 includes selected quotes for each theme across different professional roles of content validators and different survey questions.

### Key Themes Prominent in Participants’ Content Validation Feedback

1. **Reflection on the measurement of key constructs and the aim of the survey:** Content validators provided numerous suggestions regarding how questions endeavored to measure involvement with the referral process and the way perceptions can be defined and shared.
2. **Grammar suggestions:** Content validators provided several purely grammatical suggestions to improve how questions are structured and perceived by readers.
3. **Suggestions for better organization of questions:** A number of suggestions were received on ways to reword and reorganize a question to provide greater clarity.
4. **Suggestions for clarity around acronyms and abbreviations:** Some commonly used/recognized acronyms (e.g., EMR for electronic medical record, SDOH for social determinants of health) were used throughout the original survey. Content validators suggested spelling out these acronyms to ensure there is no confusion or misinterpretation of acronyms.
5. **Recommendations on redundant questions:** Content validators recommended the use of enhanced skip logic to avoid redundancy in questions and improve the overall survey flow.
6. **Recommendations on additional response possibilities for questions:** Content validators also provided feedback regarding alternative answer choices, such as additional timeframes and the use of yes/no answer options where possible.

## DISCUSSION

This study presents the development and content validation of a PCE survey by a panel of experts in rural Appalachian NC. The interdisciplinary team of researchers endeavored to craft an evidence-based process for content validating the survey, considering the unique rural Appalachian perspective and the nuances and various meanings of certain types of clinical, primary care, and referral-related terminology. The process aimed to assess the relevance of the survey while actively seeking opportunities for improvement. The high CVI–degree of relevance scores provided for each individual survey question and measure indicate that the overall approach and survey aim resonates with local clinicians and individuals with expertise in SDOH. In other words, the survey was appropriate and useful, as found by content experts. However, several key enhancements were made following validator feedback, including the following:

1. Questions requesting participants indicate their level of agreement with a set of ideas or concepts were re-written to include clear declarative statements.
2. Response options for questions asking for a ranking of confidence and comfortability with performing referral-related tasks were re-labeled to echo the same language used in the initial question itself.
3. Questions referring to “low income” were reframed to address provider and clinic staff members’ referral practices for families of all socio-economic backgrounds.
4. Questions requiring respondents to rank their confidence in services and policies were augmented to include a timeframe (current v. future).
5. Questions including acronyms were revised to include the full text/definition of acronyms.
6. Questions were revised to apply to all primary care providers more comprehensively, removing language specific to a pediatric population.

## IMPLICATIONS

This study has several notable implications for future research centered on survey design and content validation in primary care settings. The findings highlight the importance of inclusive language, declarative statements, temporality (use of clear timeframes), and consistency in survey format and design (such as headers used for ranking being tied directly to questions). This study and the final survey instrument also lay the foundation for collaborative, collective-impact initiatives that are directly informed by the survey findings. In the short term, the final, content-validated survey will be used for formative, collaborative data collection efforts within a small rural health network led by the research team and composed of local providers practicing in AR counties of NC. Findings from the survey will be used to (1) identify general trends in providers’ and clinic staff’s engagement and burnout; (2) identify strengths and gaps in current referral processes based on SDOH within each participating clinic and the rural health network at large; and (3) identify trends in provider and clinic staff self-efficacy and confidence in making referrals based on SDOH. Findings will inform evidence-based quality improvement projects to strengthen and streamline the referral process from primary care clinics and providers, address provider burnout, and identify strategies to increase, strengthen, and enhance provider and staff self-efficacy and confidence in making referrals based on SDOH. Long-term goals include expanded data collection efforts that cast a wider net within the AR of NC and to other parts of Appalachia.

Several major strengths of this initial content validation effort are worth noting: To the authors’ knowledge, this is the first study of its kind to develop and content validate a comprehensive survey instrument that assesses engagement and burnout, referral practices, and self-efficacy and confidence in making referrals based on SDOH among PCPs and clinic staff in rural Appalachia. Further, the inclusion of the intended audience in the content validation process enhances community ownership and maximizes applicability of the final survey.

A few limitations should also be noted: First, in an effort to engage healthcare providers and researchers with local and subject-matter expertise, the research team recruited content validators using convenience sampling. Participants were asked to provide their professional expertise and candid feedback regarding individual questionnaire items. Limitations of this approach include the inherent sampling bias and social desirability bias present when recruiting a group of local individuals. Second is the manner in which questions asked of the respondents were framed to focus on the validity of individual questions. Third, the small sample size and focus on a rural Appalachian context may limit generalizability of these findings to other populations. However, nesting this effort within the context of the specific target populations required focused expertise that may inform other efforts to explore and understand SDOH-related referrals and PCE in other communities in rural Appalachia.

**SUMMARY BOX**

**What is already known about this topic?**

Referrals to services based on social determinants of health (SDOH) are a critical component of strengthening health systems through the mitigation of unmet health-related social needs (e.g. education, unemployment, food insecurity, housing insecurity, mental health).

**What is added by this report?**

High content validity index scores provided for each individual survey question and measure in this content validation study indicate the overall approach and survey aim resonates with local clinicians and individuals with expertise in SDOH in rural Appalachian North Carolina.

**What are the implications for future research?**

This study and the final survey instrument lay the foundation for collaborative, collective impact initiatives that are directly informed by the survey findings.

## Figures and Tables

**Table 1:** Content Validity Index (CVI)/Degree of Relevance Results

Survey Questions	Mean	Min	Max
**Q1. What is/are your position(s) at the clinic(s)/center(s) where you work? Please select all that apply**.	3.7	3	4

**Q2. Are you involved in referral services for patients/clients?**	3.9	3	4

**Q3. In the past 3 months, have you observed any ways to improve the referrals to other community services based on Social Determinants of Health?**	4	4	4

**Q4. How comfortable/confident are you with the following?**	4	4	4
a) Providing medical care to low income children			
b) Inquiring about financial and related social needs			
c) Referring families for financial and related social needs			

**Q5. How often do you routinely ask low-income parents about the following?**	3.75	3	4
a) parent educational status			
b) parent employment status			
c) parent mental health			
d) need for childcare			
e) transportation barriers			
f) food insecurity			
g) housing insecurity			
h) utilities/heating insecurity			

**Q6. Within the past 12 months, have you referred a low-income family to any of the following community resources?**	3.86	3	4
a) employment/job search services (i.e. NCJobs)			
b) adult educational services/job training programs			
c) adult mental health providers (i.e. general and specific mental health)			
d) childcare centers/providers (i.e. Children’s Council)			
e) chronic disease management (i.e. diabetes, obesity, weight management, disordered eating)			
f) Head Start sites or other preschool programs for early childhood development (CDSA)			
g) transportation assistance (local transportation system, Medicaid Transportation, etc.)			
h) local food pantries/private charities (Hunger and Health Coalition, Harvest House Baby Pantry, Boone’s Little Free Pantry) (7)			
i) public food assistance (i.e. WIC, School Lunch, Supplemental Nutrition Assistance Program)			
j) public health insurance enrollment assistance (e.g., Medicaid, State Children’s Health Insurance Program)			
k) prenatal and postnatal support (i.e. birthing and breastfeeding classes, High Country Doulas, postnatal classes and support groups, Family Connects)			

**Q7. Within the past 12 months, if you have referred a low-income family to any of the following community resources, how often?**	3.86	3	4
a) employment/job search services (i.e. NCJobs)			
b) adult educational services/job training programs			
c) adult mental health providers (i.e. general and specific mental health)			
d) childcare centers/providers (i.e. Children’s Council)			
e) chronic disease management (i.e. diabetes, obesity, weight management, disordered eating)			
f) Head Start sites or other preschool programs for early childhood development (CDSA)			
g) transportation assistance (local transportation system, Medicaid Transportation, etc.)			
h) local food pantries/private charities (Hunger and Health Coalition, Harvest House Baby Pantry, Boone’s Little Free Pantry)			
i) public food assistance (i.e. WIC, School Lunch, Supplemental Nutrition Assistance Program)			
j) public health insurance enrollment assistance (e.g., Medicaid, State Children’s Health Insurance Program)			
k) prenatal and postnatal support (i.e. birthing and breastfeeding classes, High Country Doulas, postnatal classes and support groups, Family Connects)			
l) housing services (Hospitality House)			
m) utility assistance programs (NC LEAP)			

**Q8. What is your general perception of each of the following services?**	3.57	3	4
a) employment/job search services (i.e. NCJobs)			
b) adult educational services/job training programs			
c) adult mental health providers (i.e. general and specific mental health)			
d) childcare centers/providers (i.e. Children’s Council)			
e) chronic disease management (i.e. diabetes, obesity, weight management, disordered eating)			
f) Head Start sites or other preschool programs for early childhood development (CDSA)			
g) transportation assistance (local transportation system, Medicaid Transportation, etc.)			
h) local food pantries/private charities (Hunger and Health Coalition, Harvest House Baby Pantry, Boone’s Little Free Pantry)			
i) public food assistance (i.e. WIC, School Lunch, Supplemental Nutrition Assistance Program)			
j) public health insurance enrollment assistance (e.g., Medicaid, State Children’s Health Insurance Program)			
k) prenatal and postnatal support (i.e. birthing and breastfeeding classes, High Country Doulas, postnatal classes and support groups, Family Connects)			
l) housing services (Hospitality House)			
m) utility assistance programs (NC LEAP)			

**Q9. How comfortable/confident are you with making referrals to the following?**	3.86	3	4
a) employment/job search services (i.e. NCJobs)			
b) adult educational services/job training programs			
c) adult mental health providers (i.e. general and specific mental health)			
d) childcare centers/providers (i.e. Children’s Council)			
e) chronic disease management (i.e. diabetes, obesity, weight management, disordered eating)			
f) Head Start sites or other preschool programs for early childhood development (CDSA)			
g) transportation assistance (local transportation system, Medicaid Transportation, etc.)			
h) local food pantries/private charities (Hunger and Health Coalition, Harvest House Baby Pantry, Boone’s Little Free Pantry)			
i) public food assistance (i.e. WIC, School Lunch, Supplemental Nutrition Assistance Program)			
j) public health insurance enrollment assistance (e.g., Medicaid, State Children’s Health Insurance Program)			
k) prenatal and postnatal support (i.e. birthing and breastfeeding classes, High Country Doulas, postnatal classes and support groups, Family Connects)			
l) housing services (Hospitality House)			
m) utility assistance programs (NC LEAP)			

**Q10. During the past three months, have parents/caregivers brought up an issue regarding financial and/or related social needs about which you felt you did not have sufficient information to adequately address their concerns?**	3.86	3	4

**Q11. If yes, what was the issue(s)? Please briefly describe**	3.67	3	4

**Q12. How would you characterize your participation in referrals to services based on Social Determinants of Health?**	3.43	2	4

**Q13. Could you briefly describe your observation in ways to improve the referrals to services based on Social Determinants of Health?**	3.71	3	4

**Q14. How would you characterize your clinic’s participation in referrals to services based on Social Determinants of Health?**	4	4	4

**Q15. If your clinic is at all active in referrals to services based on Social Determinants of Health, could you briefly describe some of the activities?**	3.86	3	4

**Q16. How strongly do you agree or disagree with the following?**	4	4	4
a) It is my job to refer parents to available clinic and community resources when financial hardship and related social needs are identified			
b) It is feasible to screen for family financial and related social needs routinely at health care visits			
c) It is important to screen for family financial and related social needs routinely at health visits			
d) Addressing financial and related social needs at health care visits can have a positive impact on a child’s life			
e) I am well prepared to specifically address my patients’ families’ financial and related social needs			
f) I am effective in assisting families with their financial and related social needs			

**Q17. How strongly do you agree or disagree with the following?**	3.57	2	4
a) Increase access to health care for all children			
b) Increase the minimum wage			
c) Increase availability of subsidized pre-kindergarten/Head Start programs			
d) Expand tax credits for low-income families (e.g., the Earned Income Tax Credit program)			
e) Increase availability of affordable housing			
f) Increase availability of public food assistance programs (e.g. WIC, school lunch, food stamps (SNAP))			
g) Expand availability of adult education/job training programs			

**Q18. How strongly do you agree or disagree with the following?**	3.57	2	4
a) It is the responsibility of the government to take care of people who can’t take care of themselves			
b) The government should help more needy people even if it means going deeper in debt			
c) The government should guarantee every citizen enough to eat and a place to sleep			
d) The government should guarantee every child enough to eat and a place to sleep			
e) The government should guarantee every citizen has health insurance			
f) The government should guarantee every child has health insurance			

**Q19. How much do you think government policies and programs can do to reduce poverty in this country?**	3.43	2	4

**Q20. Overall, I am satisfied with my current job:**	3.57	2	4

**Q21. Using your own definition of “burnout”, please circle one of the answers below:**	3.71	2	4

**Q22. My professional values are well aligned with those of my department leaders:**	3.57	2	4

**Q23. The degree to which my care team works efficiently together is:**	3.71	3	4

**Q24. I feel a great deal of stress because of my job:**	3.57	2	4

**Q25. The amount of time I spend on the electronic medical record (EMR) at home is:**	3.83	3	4

**Q26. Sufficiency of time for documentation is:**	3.71	3	4

**Q27. Which number best describes the atmosphere in your primary work area?**	3.86	3	4

**Q28. My control over my workload is:**	3.86	3	4

**Q29. The EMR adds to the frustration of my day**.	3.86	3	4

**Q30. What county do you practice in?**	3.71	3	4

**Q31. What facility/clinic do you practice in? Include all facilities/clinic where you work**.	3.86	3	4

**Q32. What is your role in this organization?**	3.57	3	4

**Q33. What is your gender?**	N/A	N/A	N/A

**Q34. What is your race?**	N/A	N/A	N/A

**Q35. What is your ethnicity?**	N/A	N/A	N/A

**Q36. Your sexual orientation is:**	N/A	N/A	N/A

**Q37. How long have you been working at this organization?**	N/A	N/A	N/A

**Table 2:** Selected Comments by Theme and Survey Question

Theme/Comment Type	Survey Question	Comment & Content Validator Role
**Measurement of Key Constructs Related to Referrals and Patient Services**	Q2. Are you involved in referral services for patients/clients?	*“You might consider asking more about this involvement. Is involvement simply putting in a referral order, delegating to another staff member, or actively contacting another entity to make the referral.”*–Nurse Practitioner (NP)
	Q8. What is your general perception of each of the following services?	*“Perception- add definition or examples afterward for clarity. Maybe general perceptions (ideas, thoughts, opinions, attitudes).”* -Academic (faculty/researcher)
	Q19. How much do you think government policies and programs can do to reduce poverty in this country?	*“Is this based on current or future policies? Perhaps base it on confidence in the current policies.”* –Administrator/Practice Manager
**Grammar Suggestions**	Q18. How strongly do you agree or disagree with the following? a) It is the responsibility of the government to take care of people who can’t take care of themselves b) The government should help more needy people even if it means going deeper in debt c) The government should guarantee every citizen enough to eat and a place to sleep d) The government should guarantee every child enough to eat and a place to sleep e) The government should guarantee every citizen has health insurance f) The government should guarantee every child has health insurance	*“The clauses are worded in imperative form, not declarative form. It's jarring to agree with a command.”* –Administrative, Staff Supervisor
	Q19. How much do you think government policies and programs can do to reduce poverty in this country?	*“Delete "do to". These questions don't seem to be working toward the goals of the survey except to see if the survey taker thinks addressing SDOH is important?”* –Administrative, Staff Supervisor
**Question Re-organization & Rephrasing**	Q7. Within the past 12 months, if you have referred a low-income family to any of the following community resources, how often?	*“Rephrase: HOW OFTEN, in the past 12 months, have you referred a low-income family to any of the following community resources”* – Academic (faculty/researcher)
	Q9. How comfortable/confident are you with making referrals to the following?	*“I like that you included "confident" along with "comfortable", but I did not focus on "confident" when first responding since this word was not included a second time right above the items.”* – Nurse Practitioner (NP)
	Q10. During the past three months, have parents/caregivers brought up an issue regarding financial and/or related social needs about which you felt you did not have sufficient information to adequately address their concerns?	*“This is two questions in one- I would recommend asking one question as "in the past 3 months, have parents/caregivers brought up an issue regarding financial needs which you felt..." And then a separate question about social needs. Therefore you can describe what social needs you are referring to and also the respondent can more directly respond to financial AND social needs because they may be different.”* – Academic (faculty/researcher)
	Q33. What is your gender?	*“Another way to describe this is listed below: What is your self-identified gender? (1) Man; (2) Woman; (3) Non-binary/Gender nonconforming; (4) Prefer not to disclose; (5) prefer to self- describe: ____________.”* – Academic (faculty/researcher)
**Clarity Around Acronyms and Abbreviations**	Q29. The EMR adds to the frustration of my day.	*“I would spell out EMR. I know many readers may know this acronym, but just to be certain.”* - Academic (faculty/researcher)
**Redundancy and Duplication**	Q11. If yes, what was the issue(s)? Please briefly describe. Question follows Q10. During the past three months, have parents/caregivers brought up an issue regarding financial and/or related social needs about which you felt you did not have sufficient information to adequately address their concerns?	*“Love qualitative data- I would recommend using skip logic so that individuals who answered no do not need to see the "if yes, what was the issue" question.”* – Academic (faculty/researcher)
**Additional Response Options**	Q7. Within the past 12 months, if you have referred a low-income family to any of the following community resources, how often?	*“I would suggest adding quarterly to the list. I marked monthly but really made those referrals less often than monthly.”* – Nurse Practitioner (NP)
